# Low-density lipoprotein cholesterol and survival in pulmonary arterial hypertension

**DOI:** 10.1038/srep41650

**Published:** 2017-02-15

**Authors:** Grzegorz Kopeć, Marcin Waligóra, Anna Tyrka, Kamil Jonas, Michael J. Pencina, Tomasz Zdrojewski, Deddo Moertl, Jakub Stokwiszewski, Paweł Zagożdżon, Piotr Podolec

**Affiliations:** 1 Department of Cardiac and Vascular Diseases, Faculty of Medicine, Jagiellonian University Medical College, John Paul II Hospital in Krakow, Krakow, Poland; 2 Duke Clinical Research Institute, Duke University, Durham, NC, USA; Department of Biostatistics and Bioinformatics, Duke University, Durham, NC, USA; 3 Department of Arterial Hypertension and Diabetology, Medical University of Gdansk, Gdansk, Poland; 4 Department-Centre of Monitoring and Analyses of Population Health, National Institute of Public Health — National Institute of Hygiene, Warsaw, Poland; 5 Third Department of Internal Medicine, University Hospital St. Poelten, St. Poelten, Austria; 6 Department of Hygiene and Epidemiology, Medical University of Gdańsk.

## Abstract

Low-density lipoprotein cholesterol(LDL-C) is a well established metabolic marker of cardiovascular risk, however, its role in pulmonary arterial hypertension (PAH) has not been determined. Therefore we assessed whether LDL-C levels are altered in PAH patients, if they are associated with survival in this group and whether pulmonary hypertension (PH) reversal can influence LDL-C levels. Consecutive 46 PAH males and 94 females were age matched with a representative sample of 1168 males and 1245 females, respectively. Cox regression models were used to assess the association between LDL-C and mortality. The effect of PH reversal on LDL-C levels was assessed in 34 patients with chronic thromboembolic pulmonary hypertension (CTEPH) undergoing invasive treatment. LDL-C was lower in both PAH (2.6 ± 0.8 mmol/l) and CTEPH (2.7 ± 0.7 mmol/l) patients when compared to controls (3.2 ± 1.1 mmol/l, p < 0.001). In PAH patients lower LDL-C significantly predicted death (HR:0.44/1 mmol/l, 95%CI:0.26–0.74, p = 0.002) after a median follow-up time of 33(21–36) months. In the CTEPH group, LDL-C increased (from 2.6[2.1–3.2] to 4.0[2.8–4.9]mmol/l, p = 0.01) in patients with PH reversal but remained unchanged in other patients (2.4[2.2–2.7] vs 2.3[2.1–2.5]mmol/l, p = 0.51). We concluded that LDL-C level is low in patients with PAH and is associated with an increased risk of death. Reversal of PH increases LDL-C levels.

Pulmonary arterial hypertension (PAH) is a severe disease with high mortality^1^,^2^,^3^. Till now some prognostic factors, including clinical signs of right heart failure, impaired exercise capacity, right ventricular dysfunction and impaired pulmonary hemodynamics have been associated with poor outcome^1^,^2^,^3^. Recently, more focus has been placed on the prognostic role of alterations in the glucose and lipid metabolism^4^,^5^,^6^,^7^. Low high-density lipoprotein cholesterol (HDL-C) in PAH patients has been associated with worse clinical outcomes and higher mortality. This finding was independent of cardiovascular risk factors, insulin resistance and severity of the disease. It was hypothesized that anti-inflammatory properties of HDL-C, its ability to enhance prostacyclin half-life and its protective role in endothelium could explain its impact on prognosis in PAH^4^,^5^,^6^,^7^.

Low-density lipoprotein cholesterol (LDL-C) is an established marker of cardiovascular risk^8^. While high LDL-C levels are associated with worse prognosis in the general population, there are some populations with chronic diseases including diabetes, heart failure^9^, chronic kidney disease^10^,^11^ and rheumatoid arthritis^12^,^13^ where low LDL-C levels have been linked to increased mortality.

In PAH, low LDL-C levels might accelerate disease progression by several mechanisms including exacerbation of inflammation^14^,^15^ and direct effects on the arterial wall^16^. In fact, we have recently shown that low LDL-C levels are associated with increased stiffness of pulmonary arteries^16^. Conversely, as observed in other diseases, chronic inflammation^14^,^15^, hepatic congestion^17^ and low nutritional status^18^,^19^ might be associated with reduced LDL-C levels in individuals with PAH. If this is true, clinical improvement of a patient could lead to normalization of LDL-C levels. However, data regarding the role of LDL-C in PAH is scarce.

We therefore investigated whether LDL-C levels are altered in PAH patients and whether they are associated with survival in this group. Additionally, we evaluated whether reversal of pulmonary hypertension (PH) can influence LDL-C levels. For this purpose we studied another group of patients with chronic thromboembolic pulmonary hypertension (CTEPH) which is the only potentially curable type of PH.

## Results

### PAH population

The PAH sample included 140 patients. The majority (n = 109; 78%) of them were newly diagnosed and treatment naive at inclusion. The remaining patients were already on monotherapy with sildenafil (n = 14; 10%), endothelin receptor antagonists (n = 13; 9.3%), or prostanoids (n = 2; 1.4%) or on combination therapy with endothelin receptor antagonists and sildenafil (n = 2, 1.4%). At enrollment 15 patients used atorvastatin at mean dose of 20.7 ± 8.8 mg and 13 patients used simvastatin at mean dose of 17.9 ± 4.3 mg. During the observation period the doses did not changed significantly, respectively 18.7 ± 11.3 mg for atorvastatin (p = 0.59) and 15.4 ± 12.3 mg (p = 0.5) for simvastatin. Diabetes was present in 11 (7.8%) patients, hypertension in 35 (25%) patients, obesity in 18 (13%) patients and overweight in 38 (27%) patients. Eight (5.7%) patients were active smokers, no patient declared alcohol abuse. The mean creatinine level was 85.9 ± 42.9 μmol/l, mean aspartate transaminase (AST) 28.4 ± 12.8 U/l, and mean alanine transaminase (ALT) 29.7 ± 29.9 U/l. Seventeen (12%) patients were actively treated due to thyroid function abnormalities, however at enrollment all patients had thyroid stimulating hormone (TSH) values within normal range. Twenty two (16%) patients had higher education and 38 (27%) patients were still working full-time at enrollment. As most (n = 99; 70.7%) patients were in World Health Organization functional class (WHO-FC) III their daily activities were limited. The patients were not advised any specific dietary recommendations. Clinical, demographic, laboratory and hemodynamic data of PAH population are presented in Table 1.

### Comparison with age and sex-matched controls

LDL-C was stable during 3 month follow-up: 2.57 ± 0.88 mmol/l at enrollment and 2.59 ± 0.98 mmol/l at follow-up; p = 0.67. As compared to controls it was lower in PAH women, men and patients who did not use statins (Table 2). LDL-C levels were similar in the subgroups of idiopathic PAH (IPAH), PAH associated with connective tissue disease (CTD-PAH), and PAH associated with congenital heart disease (CHD-PAH) (2.4[1.7–3.0] vs 2.8 [2.1–3.6] vs and 2.6 [2.1–3.0] mmol/l, respectively, p = 0.83). Sex–specific comparisons of other cardiovascular risk factors between PAH patients and age-matched controls are presented in Table 2.

### Clinical characteristics and LDL-C levels

The associations of age, sex, aspartate aminotransferase (AST), alanine aminotransferase (ALT), creatinine, cardiovascular risk factors, markers of PAH severity and LDL-C levels are presented in Table 3. In univariate linear regression analysis triglycerides (TG), WHO-FC class and sex were associated with LDL-C level, however in a multiple regression model [R^2^ = 0.26, F(2, 86) = 15.2, p < 0.001] including all variables given in Table 3 significant relationship with LDL-C remained only for TG (beta coefficient [standard error] = 0.46 [0.09], p < 0.001) and WHO-FC (β coefficient [standard error] = −0.27 [0.09], p = 0.005). In a subgroup of 108 patients in whom high-sensitivity C-reactive protein (hsCRP) was available (patients recruited after January 2011) we found a significant correlation (r = −0.24 and p = 0.01) between this marker and LDL-C level.

### LDL-C for the prediction of death in PAH

**Figure 1:**
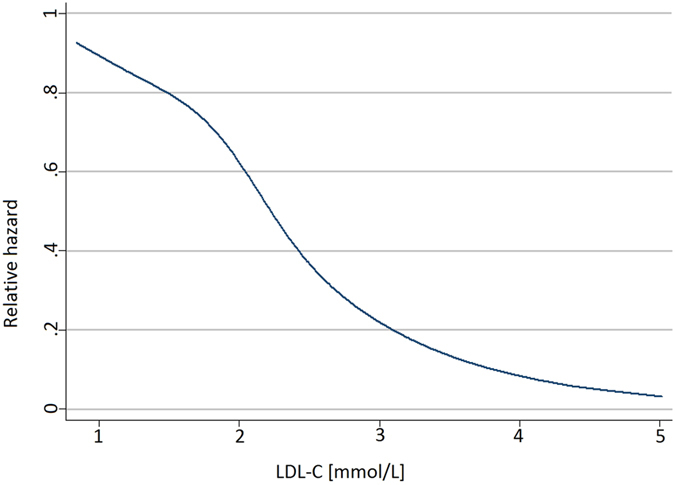
Association between low-density lipoprotein cholesterol (LDL-C) levels and relative hazard for death in patients with pulmonary arterial hypertension.

In the PAH sample, 32 patients died after a median follow-up time of 33^20^ months. Spline analysis revealed the association between LDL-C levels and survival to be linear (Fig. 1), allowing to model LDL-C in this way. Lower LDL-C levels were significantly associated with all-cause mortality in univariable analysis (HR: 0.44 per 1 mmol/l, 95% CI: 0.26–0.74, p = 0.002, Table 4) and after hierarchical adjustment for risk factors (HR in fully-adjusted model: 0.18 per 1 mmol/l, 95% CI: 0.07–0.47, p < 0.001, Table 4). Similar results were found when only patients who did not use statins were analysed (HR in fully-adjusted model: HR = 0.19 per mmol/l, 95% CI = 0.06–0.46, p < 0.001).

### LDL-C changes after treatment in CTEPH patients

The total CTEPH population in our registry consisted of 34 patients (14 males) aged 64.5 [52.0–71.0] years. They were in WHO FC II (n = 4), III (n = 28), and IV (n = 2) on presentation. Similar to the PAH group, the CTEPH patients had lower LDL-C levels than the age and sex-matched control patients from the NATPOL study (2.7 ± 0.7 mmol/l vs. 3.2 ± 1.1 mmol/l, p < 0.001). In 21 patients we had data on LDL-C and hemodynamics at baseline and follow-up. The reversed CTEPH (rCTEPH) group consisted of 11 patients (4 males) aged 47.0 [42.0–65.0] years, in whom by definition mPAP decreased at least by ≥10 mmHg and to <30 mmHg. In this group mPAP decreased from 40[31–50] to 23[19–27] mmHg; p = 0.015, pulmonary vascular resistance (PVR) from 8.7[2.5–11.5] to 2.6[2.1–5.3] WU, p = 0.015, right atrial pressure (RAP) from 6.5 [4–9] to 5[2–14] mmHg; p = 0.1, while cardiac index (CI) increased from 2.1 [1.7–2.5] to 2.5 [2.0–2.7]; p = 0.045. The nonreversed (nrCTEPH) group consisted of 10 patients (5 males) aged 66.0 [61.0–77.0] years in whom by definition there was no significant decrease of mPAP during observation. In this group hemodynamic parameters were similar at baseline and at follow-up (mPAP 50.5[46–56] vs 47[43–51] mmHg; p = 0.85, PVR 13.5[13.0–14.7] vs 13.1 [10.7–13.7] WU; p = 0.80, RAP 3[1–4] vs 5[2–14] mmHg; p = 1.0, CI 1.8[1.7–1.9] vs 1.9[1.7–2.3] l/min/m^2^; p = 1.0).

**Figure 2:**
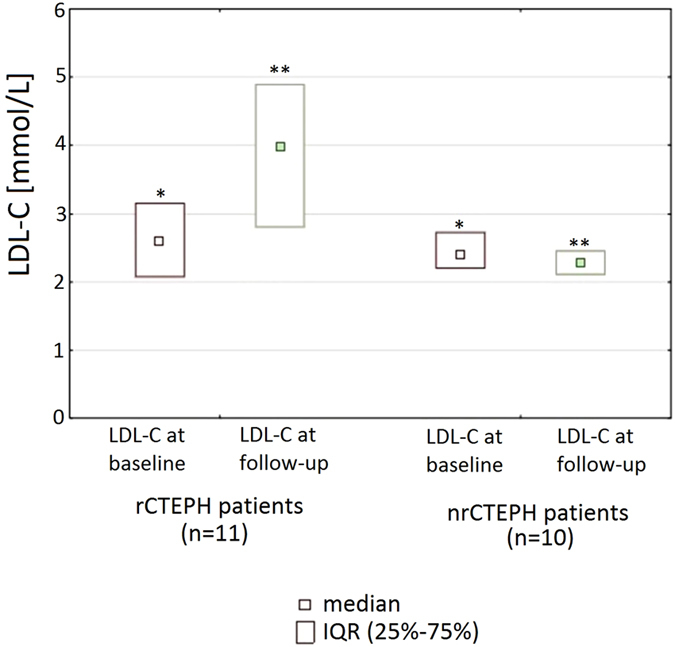
Low-density lipoprotein cholesterol (LDL-C) levels at baseline and follow-up in the reversed (rCTEPH) and non-reversed chronic thromboembolic pulmonary hypertension (nrCTEPH) patients. *p = 0.6 for the comparison of baseline data between rCTEPH and nrCTEPH group, **p = 0.005 for the comparison of follow-up data between rCTEPH and nrCTEPH group.

rCTEPH patients were treated with pulmonary endarterectomy (PEA, n = 4) or balloon pulmonary angioplasty (BPA, 5.0 [2.0–7.0] sessions per patient; n = 7) while nrCTEPH patients had monotherapy with riociguat (n = 1), sildenafil (n = 2), subcutaneous treprostinil (n = 1), one BPA session (n = 3), or a combination of sildenafil and subcutaneous treprostinil (n = 2) or subcutaneous treprostinil and one BPA session (n = 1). Follow-up time was similar in the rCTEPH and nrCTEPH group (12 [7–30] vs 18 [11–25] months, p = 0.5). During this time LDL-C increased from 2.6 [2.1–3.2] to 4.0 [2.8–4.9] mmol/l; p = 0.045 in the rCTEPH group, but remained unchanged in nrCTEPH patients: 2.4 mmol/l [2.2–2.7] vs 2.3 mmol/l [2.1–2.5], p = 1.0 (Fig. 2).

## Discussion

In the present study we show that PAH patients have decreased LDL-C levels, and that low LDL-C levels are associated with increased risk of all-cause mortality. Furthermore, we demonstrate that in CTEPH patients significant improvement of hemodynamic parameters is associated with reversal of decreased LDL-C levels.

Metabolic changes as reflected by decreased levels of HDL-C and increased insulin resistance have been described previously in the PAH population^4^,^5^,^6^,^7^. Low HDL-C in PAH patients has been associated with worse clinical outcomes and higher mortality. This finding was independent from cardiovascular risk factors, insulin resistance and severity of the disease. This phenomenon may be explained by antiinflammatory properties of HDL-C, its ability to enhance prostacyclin half-life and its protective role in endothelial dysfunction.

The phenomenon of reduced LDL-C levels has already been described in other chronic diseases such as rheumatoid arthritis^12^, cancer^20^, end-stage renal failure^11^ or chronic heart failure^21^ however, to the best of our knowledge this is the first study reporting changes in LDL-C levels and their prognostic role in PAH patients. Potential explanations for these findings include a chronic inflammatory state^22^, malnutrition^18^, and altered liver metabolism^17^.

Inflammatory cytokines are able to activate the reticuloendothelial system leading to enhanced receptor-independent clearance of LDL-C^23^. Conversely, suppression of the reticuloendothelial system leads to inhibition of LDL-C clearance^24^. Studies in rheumatoid arthritis have shown that suppression of inflammation with disease-modifying antirheumatic drugs or biological agents which target specific proinflammatory cytokines was accompanied by an increase in total cholesterol and LDL-C levels^25^,^25^. What is more, this increase was able to differentiate responders from non-responders to antirheumatic therapies^27^. An expanding body of knowledge suggests that inflammation and altered immune processes underlie both development and progression of PAH^14^,^28^. In fact we found a significant correlation between hsCRP and LDL-C in subgroup analysis of our PAH patients. However further studies are needed to fully understand the relationship between inflammation and lipid profile in this group.

As the liver plays a central role in lipid metabolism controlling both production and clearance of serum lipoproteins it seems likely that its dysfunction could contribute to altered LDL-C levels in PAH. Right heart dysfunction can lead to congestive hepatopathy, which is related to right atrial pressure and the degree of tricuspid regurgitation^17^. In fact, in our study we demonstrated that significant improvement of pulmonary hemodynamics in CTEPH patients was associated with normalization of LDL-C plasma levels. However, the exact pathophysiological pathways involved still need to be identified, since LDL-C normalization might not only be a direct consequence of hemodynamic improvement but also may result from improvement of the general condition of the patient^29^,^30^.

Low LDL-C levels have also been found in malnourished patients with chronic heart failure^9^. Accordingly, malnutrition could also contribute to the observed reduction of LDL-C in PAH patients. However, in our cohort none of the available markers of malnutrition such as body mass index (BMI) were associated with LDL-C level.

Several concomitant disorders and lifestyle interventions can influence LDL-C level^31^. Dietary changes, reduction of excessive body weight and increase in habitual physical activity may lead to lowering LDL-C concentration^32^. However we do not think that these lifestyle factors had significant impact on LDL-C level in our population. First, no specific recommendations on dietary patterns or weight reduction has been given to patients due to lack of scientific evidence. Second, the ability to increase of physical activity is limited in PAH patients due to exertional dyspnea. Third, most of our patients were within reference values for BMI and we did not find any correlation between LDL-C and BMI. Renal or hepatic impairment as well as thyroid abnormalities can also influence LDL-C level. In our patients, however, we did not find any associations between markers of these disorders (ALT, AST, creatinine) and LDL-C level. Thyroid abnormalities when present were well controlled. An important modulator of LDL-C concentration is the use of statins. Therefore, when we compared LDL-C levels to controls we analyzed separately all PAH patients and PAH patients who did not use statins. In both comparisons LDL-C levels were significantly decreased in PAH group. We monitored the use of statins at follow-up and did not find significant change in the number of patients who used these drugs as well as doses of statins in individual patients. In multivariable analysis we considered the use of statins as a potential confounder.

Our study suggests that traditional interpretation of hypercholesterolemia as a risk factor for increased mortality may not apply in PAH population. Instead, our finding of the deleterious signal of low LDL-C levels in PAH corresponds with the concept of cholesterol paradox and reverse epidemiology whereby lower levels of traditional risk factors are associated with worse prognosis^22^. The evidence of a survival advantage associated with higher cholesterol levels has been provided for several populations with debilitating disorders such as heart failure^9^, rheumatoid arthritis^12^, acute myocardial infarction^33^,^34^ and in the elderly^35^,^36^.

The mechanism of interaction between LDL-C and clinical outcome has not been investigated in PAH. In other populations the importance of lipoproteins in down-regulation of the inflammatory immune response^9^ and prevention of malnutrition^18^ has been claimed. Lipids and lipoproteins can modulate inflammatory markers^22^,^37^,^38^,^39^,^40^. Cholesterol can bind and neutralize endotoxin and liposaccharide components of infectious agents, thereby down-regulating the inflammatory state, which has been linked to progression of PAH^14^,^28^,^41^. Other data from experimental studies suggest that the effects of LDL-cholesterol on the arterial wall may be different in the pulmonary and systemic circulation. In rabbits, it has been demonstrated that hypercholesterolemia can increase pulmonary artery relaxation in response to methacholine^42^. Another experimental study^43^ in rats with monocrotaline induced PAH showed that high-fat diet and hypercholesterolemia was associated with better prognosis as compared with standard diet. We have recently shown that low LDL-C levels, together with BMI < 25 kg/m^2^ and higher pulmonary artery pressure are independent determinants of increased pulmonary artery stiffness and together explain most of the variation in pulmonary artery pulse wave velocity^16^. The different role of LDL-C in pulmonary circulation disease compared to the systemic circulation could also have contributed to the negative results of studies with statins in PAH patients^44^.

Our data suggests a strong linear relationship between LDL-C levels and mortality with a 2- to 5-fold increase in risk of death for every 1 mmol/l decrease in LDL-C. We cannot exclude the possibility that low LDL-C do not have any direct pathophysiologic connection to mortality and is only a consequence of the severity of PAH or malnutrition, which are also associated with poor survival. However, LDL-C levels were associate with all-cause mortality independently from markers of PAH severity – WHO FC and malnutrition - low BMI. Therefore, our data suggests that the prognostic impact of LDL-C in PAH cannot be fully explained by PAH severity or malnutrition, thereby supporting its potential role as an independent risk marker in PAH. Nevertheless, whether low LDL-C levels represent a modifiable risk marker in PAH or just an epiphenomenon with no direct pathophysiological impact on prognosis still needs to be elaborated.

Our study has several limitations. First, our data cannot provide explanations on the mechanisms by which low LDL-C levels influence survival in PAH patients. However, some plausible hypotheses can be proposed from our data for further studies. Second, as this is an observational study, we cannot establish a cause-and-effect relationship between LDL-C and outcome. Third, we could not ascertain the cause of death and compare the impact of low LDL-C on cardiovascular versus non-cardiovascular mortality or cardiovascular morbidity. Additionally we did not assess specifically dietary patterns of our patients which could have influenced LDL-C level. We also have not given the patients any specific advice on nutrition. This was mainly due to the fact that currently there is no evidence on the proper diet in patients with PAH.

Our study has several important strengths. First, the findings of LDL-C level alterations in the PAH population and their prognostic importance are novel and have not been previously reported. Second, as a control group we used a large representative sample of adults. Third, by using interventional and surgical treatment of CTEPH as a model for reliable, rapid improvement of hemodynamics, we could elaborate the dynamic relationship between LDL-C levels and severity of pulmonary hypertension beyond simple associations at baseline.

In summary, LDL-C levels are low in patients with PAH and are associated with an increased risk of death. Reversal of pulmonary hypertension in CTEPH patients increases low LDL-C levels. Future studies are necessary to gain more insights into the underlying pathophysiology of these findings and their role in the management of PAH.

## Methods

### Patient population

Consecutive patients were recruited from the PH Program at the John Paul II Hospital (Krakow, Poland) between February 2009 and January 2015. Adult patients diagnosed with PAH except portopulmonary PAH were eligible for the study. PH was defined as an increase in mPAP ≥25 mmHg at rest as assessed by right heart catheterization^3^. PAH was defined as precapillary PH (pulmonary artery wedge pressure ≤15 mmHg) with PVR > 3 Wood units in the absence of other causes of precapillary PH such as lung diseases, CTEPH or other rare diseases^3^. As a control group we chose a representative sample of 2413 Polish residents aged 18–79 years from the NATPOL 2011 Survey, a cross-sectional observational study aimed to assess the prevalence and control of cardiovascular disease risk factors in Poland^45^. All-cause mortality was ascertained by data collection from the Department of Nationals’ and Foreigners’ Affairs and through phone follow-up. The observation period started at the first assessment of the patient in our center and extended until January 2016. We also studied patients with CTEPH from our centre to see whether PH reversal can change LDL-C levels. For this purpose, we formed two subgroups to be compared: CTEPH patients with a significant hemodynamic improvement (rCTEPH) by interventional (BPA), surgical (PEA) or drug treatment. The second subgroup included CTEPH patients not meeting our definition of hemodynamic improvement (nrCTEPH). Significant hemodynamic improvement was defined as a reduction of mPAP by ≥10 mmHg and to <30 mmHg. This analysis was performed only in patients without changes in statin therapy from 3 months before baseline until follow-up. CTEPH was defined as precapillary PH characterized by mismatched perfusion defects on lung scan and specific diagnostic signs for CTEPH seen by conventional pulmonary cineangiography. The diagnosis of CTEPH could have been established only after at least 3 months of effective anticoagulation^3^. The institutional ethics committee (Komisja Bioetyczna - Okregowa Izba Lekarska w Krakowie) approved the registry of patients with PAH and CTEPH in our center and the patients signed the informed consent. The study protocol conforms to the ethical guidelines of the 1975 Declaration of Helsinki.

### Clinical assessment and laboratory measurements

Clinical assessment, laboratory measurements and hemodynamic evaluation were made at the first evaluation of a patient in our center. Clinical assessment included demographic information, patient’s history, calculation of BMI, measurement of blood pressure, and N-terminal pro-brain natriuretic peptide (NT-proBNP) level, six minute walk test and assessment of the WHO FC, as well as other tests routinely performed at first evaluation of a PAH patient in our center but not analyzed in the study such as echocardiography. Obesity was defined as BMI ≥30 kg/m^2^, and overweight as BMI ≥25 kg/m^2^ and <30 kg/m^2^ 46. Hypertension was diagnosed in patients with elevated systolic or diastolic blood pressure (BP) ≥140 or 90 mmHg, respectively or patients having been prescribed a BP-lowering drugs for high BP. On the contrary patients were not classified as hypertensives when they were assuming drugs with BP-lowering effect but prescribed for other reasons than hypertension, e.g. for angina^47^. Diabetes was diagnosed based on the plasma glucose criteria of the American Diabetes Association, either the fasting plasma glucose or the 2-h plasma glucose value after a 75-g oral glucose tolerance test^48^. Active smoker was defined as having smoked at least for 1 month during the previous 12 months; all others were classified as nonsmokers^16^. Peripheral venous blood was drawn after overnight fast in all patients (PAH and CTEPH group) on the day of right heart catheterization. Plasma lipids were measured twice in every patient. In the PAH group measurements were performed at enrollment and three months after enrollment while in the CTEPH group before starting therapy and after at least three months of therapy. Blood chemistry was performed using the Cobas^®^ 6000 analyzer (Roche Diagnostics International Ltd). LDL-C, HDL-C, and TG plasma concentrations were measured directly by using colorimetric technique with PEG-esterase, PEG-oxidase and peroxidase for HDL-C and LDL-C, and with lipase and peroxidase for TG concentrations. AST and ALT assay was performed accordingly to International Federation of Clinical Chemistry and Laboratory Medicine recommendations without pyridoxal phosphate activation. hsCRP was assessed using high sensitive latex assay. Creatinine level was assessed using kinetic colorimetric assay. Hemodynamic evaluation was performed during right heart catheterization using a Swan-Ganz-Catheter. Cardiac output was assessed using the oxygen consumption method. Clinical assessment and laboratory measurements performed in the NATPOL survey control group were described in detail elsewhere^45^.

### Statistical analysis

Continuous variables were reported as mean ± standard deviation or as median (interquartile range) and categorical variables as counts and percentages. For the comparison of continuous variables between two groups we used Student’s t-test or Mann-Whitney U-test as appropriate and for categorical variables the chi-squared test. The Wilcoxon rank sum test was used for paired comparisons of continuous parameters assessed at baseline and at follow-up. For the comparison of LDL-C levels in three different groups of patients with PAH we used the Kruskal-Wallis test.

To compare patients with controls, the groups were matched for age separately in males and females. Multiple regression analysis using forward stepwise selection was used to assess significant associations between patients’ characteristics and LDL-C levels. To exclude possible multicollinearity, we used multicollinearity diagnostic statistics and examined the variance inflation factor.

To assess the relationship between LDL-C and the risk of all-cause mortality, we used Cox proportional hazards regression. To allow for flexible modeling of the association and account for potential non-linearity, we modeled LDL-C using restricted cubic splines.

Models were hierarchically adjusted for major cardiovascular risk factors and markers of severity of PAH: model 1 - unadjusted analysis; model 2: adjusted for age and sex; model 3: adjusted additionally for diabetes, BMI, statin use, hypertension, smoking; model 4 - adjusted additionally for HDL-C, TG; model 5 - adjusted additionally for WHO-FC, etiology of PAH, PAH-specific treatment at the end of observation, NT-proBNP, 6-minute walk distance, RAP, PVR, CI; model 6 - similar to model 5 but PVR was substituted with mPAP. Sample size was calculated for the comparison of LDL-C between the rCTEPH and nrCTEPH at follow-up. We expected that in the rCTEPH group the LDL-C level would increase to the values of control group and that in the nrCTEPH group LDL-C would not change. Consequently, we expected that the difference of mean LDL-C between nrCTEPH group and rCTEPH group at follow-up would be 0.74 mmol/l. For alpha level of 0.05 and beta level of 0.2 the minimal number of patients in each group was 10. When comparing several parameters in the CTEPH group before and after treatment adjustment for multiple comparisons has been done. The significance level was set at alpha level of 0.05. Statistical analysis was performed with Statistica PL software [StatSoft, Inc. (2010). STATISTICA (data analysis software system), version 9.1. Tulsa, USA www.statsoft.com] and MedCalc version 11.6.1.0 (MedCalc Software, Mariakerke, Belgium) and STATA. 14.1 (StataCorp).

## Additional Information

**How to cite this article:** Kopeć, G. *et al*. Low-density lipoprotein cholesterol and survival in pulmonary arterial hypertension. *Sci. Rep.* **7**, 41650; doi: 10.1038/srep41650 (2017).

**Publisher's note:** Springer Nature remains neutral with regard to jurisdictional claims in published maps and institutional affiliations.

## Figures and Tables

**Table 1:** Characteristics of patients with pulmonary arterial hypertension.

PAH group (n = 140)	
Age (y)	46 [28.0–62.0]
Sex (male)	46 (32%)
PAH etiology	
IPAH	63 (45%)
CTD-PAH	17 (12%)
CHD-PAH	60 (43%)
WHO FC (II/III/IV)	19 (13.6%)/99 (70.7%)/22 (15.7%)
mPAP (mmHg)	52.0 [41.0–71.0]
RAP (mmHg)	7.0 [3.0–9.0]
PAWP (mmHg)	9.0 [6.0–12.0]
Ao mean pressure (mmHg)	92.0 ± 14.2
CO (l/min)	3.1 [2.3–4.3]
CI (l/min/m^2^)	1.75 [1.4–2.4]
PVR (WU)	15.7 [9.9–22.5]
NT-proBNP (pg/ml)	619.4 [183.2–2229.0]
6MWD (m)	321.3 ± 133.3

6-MWD – 6-minute walk distance, Ao – aorta, CHD-PAH – pulmonary arterial hypertension associated with congenital heart disease, CI – cardiac index, CO – cardiac output, CTD-PAH – pulmonary arterial hypertension associated with connective tissue disease, IPAH – idiopathic pulmonary arterial hypertension, mPAP – mean pulmonary arterial pressure, NT-proBNP – N-terminal pro-brain natriuretic peptide, PAH – pulmonary arterial hypertension, PAWP – pulmonary artery wedge pressure, PVR – pulmonary vascular resistance, RAP – right atrial pressure, WHO-FC – World Health Organization functional class.

**Table 2:** Cardiovascular risk factors in patients with pulmonary arterial hypertension and age and sex-matched general population.

	PAH (n = 140)		Controls (n = 2413)		p-value	
	f (n = 94)	m (n = 46)	f (n = 1245)	m (n = 1168)	f	m
Age (years)	43.6 ± 17.2	41.6 ± 16.4	44.8 ± 27.1	42.9 ± 24.0	0.49	0.56
BMI (kg/m^2^)	23.8 ± 4.16	24.9 ± 5.2	25.9 ± 7.3	27.1 ± 6.7	<0.001	0.002
SBP (mmHg)	109.2 ± 16.9	108.9 ± 16.6	130.1 ± 30.0	136.7 ± 26.9	<0.0001	<0.0001
DBP (mmHg)	70.3 ± 10.4	70.0 ± 11.3	80.6 ± 13.1	82.4 ± 15.7	<0.0001	< 0.0001
LDL-C (mmol/l)	2.6 ± 0.8	2.3 ± 0.9	3.2 ± 1.1	3.2 ± 1.2	<0.0001	< 0.0001
LDL-C (mmol/l)[without statins]*	2.6 ± 0.8	2.3 ± 0.8	3.3 ± 1.1	3.3 ± 1.2	<0.0001	< 0.0001
HDL-C (mmol/l)	1.3 ± 0.4	1.1 ± 0.3	1.4 ± 0.4	1.2 ± 0.5	0.02	0.18
TG (mmol/l)	1.3 ± 0.7	1.3 ± 0.8	1.1 ± 0.8	1.6 ± 1.6	0.054	0.19
Glucose (mmol/l)	5.4 ± 1.7	5.5 ± 0.9	5.0 ± 1.0	5.3 ± 1.6	0.0003	0.49
Diabetes	10%	3%	5.9%	4.6%	0.17	0.88
Statin use	19%	21%	11.9%	11.2%	0.06	0.07
Hypertension	21%	32%	25%	28.2%	0.46	0.69
Active smoker	4.9%	7.2%	27.1%	35.6%	<0.0001	0.0001

BMI – body mass index, DBP – diastolic blood pressure, f – female, m – male, HDL – high-density lipoprotein, LDL – low-density lipoprotein, PAH – pulmonary arterial hypertension, SBP – systolic blood pressure, TG – triglycerides *the number of patients who had not used statins are as follows: in PAH group: 76 females and 36 males; in control group 1097 females and 1037 males.

**Table 3:** Univariate linear regression associations between different variables and low-density cholesterol level in patients with pulmonary arterial hypertension (n = 140).

Variable	β coefficient (SE)	P value
Age (years)	0.01 (0.09)	0.88
Sex (female = 0/male = 1)	−0.18 (0.08)	0.03
WHO-FC	−0.2 (0.08)	0.02
6-MWD	0.12 (0.09)	0.17
NT-proBNP	−0.09 (0.09)	0.35
AST	−0.09 (0.09)	0.32
ALT	−0.09 (0.09)	0.3
Creatinine	−0.07 (0.09)	0.38
PAH etiology		
IPAH (yes = 1/no = 0)	−0.13 (0.09)	0.13
CTD-PAH (yes = 1/no = 0)	0.12 (0.08)	0.16
CHD-PAH (yes = 1/no = 0)	0.05 (0.09)	0.56
Hemodynamic data		
mPAP	−0.01 (0.09)	0.9
CI	−0.03 (0.09)	0.69
PVR	−0.05 (0.09)	0.59
RAP	−0.18 (0.1)	0.06
Cardiovascular risk factors		
Diabetes (yes = 1/no = 0)	0.04 (0.09)	0.67
Hypertension (yes = 1/no = 0)	0.04 (0.09)	0.65
BMI	0.1 (0.09)	0.26
HDL cholesterol	0.06 (0.09)	0.51
Triglycerides	0.4 (0.08)	<0.0001
Treatment		
statins (yes = 1/no = 0)	−0.11 (0.08)	0.18
baseline PAH therapy	0.06 (0.09)	0.45

6-MWD – 6-minute walk distance, ALT – alanine aminotransferase, AST – aspartate aminotransferase, BMI – body mass index, CHD-PAH – pulmonary arterial hypertension associated with congenital heart disease, CI – cardiac index, CTD-PAH – pulmonary arterial hypertension associated with connective tissue disease, HDL-C – high density lipoprotein cholesterol, IPAH – idiopathic pulmonary arterial hypertension, mPAP – mean pulmonary arterial pressure, NT-proBNP – N-terminal pro-brain natriuretic peptide, PAH – pulmonary arterial hypertension, PVR – pulmonary vascular resistance, RAP – right atrial pressure, SE – standard error, WHO-FC – World Health Organization functional class.

**Table 4:** Cox regression model for the association between low-density lipoprotein cholesterol and mortality.

	HR (95% CI) per 1 mmol/l	p
model 1	0.44 (0.26–0.74)	0.002
model 2	0.44 (0.26–0.74)	0.002
model 3	0.44 (0.26–0.74)	0.002
model 4	0.38 (0.22–0.68)	0.0009
model 5	0.18 (0.07–0.47)	0.0005
model 6	0.18 (0.07–0.47)	0.0005

Model 1: unadjusted analysis. Model 2: analysis adjusted for age and sex. Model 3: analysis adjusted for age, sex, diabetes, body mass index (BMI), statin use, hypertension, smoking. Model 4: analysis adjusted for age, sex, diabetes, BMI, statin use, hypertension, smoking, high density lipoprotein cholesterol (HDL-C), triglycerides. Significant predictor of death were: low-density lipoprotein cholesterol (LDL-C) and HDL-C (0.24; 0.08–0.66). Model 5: Analysis adjusted for age, sex, diabetes, BMI, statin use, hypertension, smoking, HDL-C, triglycerides, World Health Organization functional class (WHO-FC), etiology of pulmonary arterial hypertension (PAH), PAH-specific treatment at the end of observation, N-terminal pro-brain natriuretic peptide (NT-proBNP), 6-minute walk distance (6-MWD), right atrial pressure (RAP), pulmonary vascular resistance, cardiac index (CI). Significant predictors of death were: LDL-C and current smoking (6.8; 1.3–35.6), NT-proBNP (1.02; 1.01–1.03), RAP (1.12; 1.02–1.25). Model 6: Analysis adjusted for age, sex, diabetes, BMI, statin use, hypertension, smoking, HDL-C, triglycerides, WHO-FC, etiology of PAH, PAH-specific treatment at the end of observation, NT-proBNP, 6-MWD, RAP, mean pulmonary artery pressure, CI. Significant predictors of death were: LDL-C and current smoking (6.8; 1.3–35.6), NT-proBNP (1.02; 1.01–1.03), RAP (1.12; 1.02–1.25).

## References

[b1] Benza R. L. . Prognostic implications of serial risk score assessments in patients with pulmonary arterial hypertension: a Registry to Evaluate Early and Long-Term Pulmonary Arterial Hypertension Disease Management (REVEAL) analysis. J Heart Lung Transplant. 34, 356–361 (2015). 25447572 10.1016/j.healun.2014.09.016

[b2] Cogswell R., Kobashigawa E., McGlothlin D., Shaw R. & De Marco T. Validation of the Registry to Evaluate Early and Long-Term Pulmonary Arterial Hypertension Disease Management (REVEAL) pulmonary hypertension prediction model in a unique population and utility in the prediction of long-term survival. J Heart Lung Transplant. 31, 1165–1170 (2012). 23062726 10.1016/j.healun.2012.08.009

[b3] Galiè N. . ESC/ERS Guidelines for the diagnosis and treatment of pulmonary hypertension: The Joint Task Force for the Diagnosis and Treatment of Pulmonary Hypertension of the European Society of Cardiology (ESC) and the European Respiratory Society (ERS): Endorsed by: Association for European Paediatric and Congenital Cardiology (AEPC), International Society for Heart and Lung Transplantation (ISHLT). Eur Respir J. 46, 903–975 (2015). 26318161 10.1183/13993003.01032-2015

[b4] Brunner N. W. . Impact of insulin resistance on ventricular function in pulmonary arterial hypertension. J Heart Lung Transplant. 33, 721–726 (2014). 24819985 10.1016/j.healun.2014.02.016

[b5] Zamanian R. T. . Insulin resistance in pulmonary arterial hypertension. Eur Respir J. 33, 318–324 (2009). 19047320 10.1183/09031936.00000508 PMC2785883

[b6] Heresi G. A., Aytekin M., Newman J., DiDonato J. & Dweik R. A. Plasma levels of high-density lipoprotein cholesterol and outcomes in pulmonary arterial hypertension. Am J Respir Crit Care Med. 182, 661–668 (2010). 20448092 10.1164/rccm.201001-0007OC PMC2937236

[b7] Zhao Q. H. . Serum high-density lipoprotein cholesterol levels as a prognostic indicator in patients with idiopathic pulmonary arterial hypertension. Am J Cardiol. 110, 433–439 (2012). 22560769 10.1016/j.amjcard.2012.03.042

[b8] Lewington S. . Blood cholesterol and vascular mortality by age, sex, and blood pressure: a meta-analysis of individual data from 61 prospective studies with 55,000 vascular deaths. Lancet. 370, 1829–1839 (2007). 18061058 10.1016/S0140-6736(07)61778-4

[b9] Charach G. . Baseline low-density lipoprotein cholesterol levels and outcome in patients with heart failure. Am J Cardiol. 105, 100–104 (2010). 20102899 10.1016/j.amjcard.2009.08.660

[b10] Bowden R. G. . Reverse epidemiology of lipid-death associations in a cohort of end-stage renal disease patients. Nephron Clin Pract. 119, c214–219 (2011). 21832847 10.1159/000329509

[b11] Peev V., Nayer A. & Contreras G. Dyslipidemia, malnutrition, inflammation, cardiovascular disease and mortality in chronic kidney disease. Curr Opin Lipidol. 25, 54–60 (2014). 24345987 10.1097/MOL.0000000000000045

[b12] Choy E. & Sattar N. Interpreting lipid levels in the context of high-grade inflammatory states with a focus on rheumatoid arthritis: a challenge to conventional cardiovascular risk actions. Ann Rheum Dis. 68, 460–469 (2009). 19286905 10.1136/ard.2008.101964

[b13] Situnayake R. D. & Kitas G. Dyslipidemia and rheumatoid arthritis. Ann Rheum Dis. 56, 341–342 (1997). 9227161 10.1136/ard.56.6.341 PMC1752397

[b14] Rabinovitch M., Guignabert C., Humbert M. & Nicolls M. R. Inflammation and immunity in the pathogenesis of pulmonary arterial hypertension. Circ Res. 115, 165–175 (2014). 24951765 10.1161/CIRCRESAHA.113.301141 PMC4097142

[b15] Soon E. . Elevated levels of inflammatory cytokines predict survival in idiopathic and familial pulmonary arterial hypertension. Circulation 122, 920–927 (2010). 20713898 10.1161/CIRCULATIONAHA.109.933762

[b16] Kopec G. . Pulmonary artery pulse wave velocity in idiopathic pulmonary arterial hypertension. Can J Cardiol. 29, 683–690 (2013). 23260799 10.1016/j.cjca.2012.09.019

[b17] Møller S. & Bernardi M. Interactions of the heart and the liver. Eur Heart J. 34, 2804–2811 (2013). 23853073 10.1093/eurheartj/eht246

[b18] Araújo J. P. . Cholesterol–a marker of nutritional status in mild to moderate heart failure. Int J Cardiol. 129, 65–68 (2008). 17643521 10.1016/j.ijcard.2007.05.026

[b19] Kawamoto A. . Relationships between nutritional status and markers of congestion in patients with pulmonary arterial hypertension. Int J Cardiol. 187, 27–28 (2015). 25828304 10.1016/j.ijcard.2015.03.354

[b20] Alexopoulos C. G. . Changes in serum lipids and lipoproteins in cancer patients during chemotherapy. Cancer Chemother Pharmacol. 30, 412–416 (1992). 1505080 10.1007/BF00689971

[b21] Gombos T. . Long-Term Survival and Apolipoprotein A1 Level in Chronic Heart Failure: Interaction With Tumor Necrosis Factor α-308 G/A Polymorphism. J Card Fail. 2016 Jun 16. doi: 10.1016/j.cardfail.2016.06.004. [Epub ahead of print]. 10.1016/j.cardfail.2016.06.004 27317841

[b22] Liu Y. . Association between cholesterol level and mortality in dialysis patients: role of inflammation and malnutrition. JAMA 291, 451–459 (2004). 14747502 10.1001/jama.291.4.451

[b23] Kruth H. S. . Macropinocytosis is the endocytic pathway that mediates macrophage foam cell formation with native low density lipoprotein. J Biol Chem. 280, 2352–2360 (2005). 15533943 10.1074/jbc.M407167200

[b24] Slater H. R., Packard C. J. & Shepherd J. Receptor-independent catabolism of low density lipoprotein. Involvement of the reticuloendothelial system. J Biol Chem. 257, 307–310 (1982). 6273431

[b25] Lee Y. H., Choi S. J., Ji J. D., Seo H. S. & Song G. G. Lipoprotein(a) and lipids in relation to inflammation in rheumatoid arthritis. Clin Rheumatol. 19, 324–325 (2000). 10941819 10.1007/pl00011174

[b26] Emery P. . IL-6 receptor inhibition with tocilizumab improves treatment outcomes in patients with rheumatoid arthritis refractory to anti-tumour necrosis factor biologicals: results from a 24-week multicentre randomised placebo-controlled trial. Ann Rheum Dis. 67, 1516–1523 (2008). 18625622 10.1136/ard.2008.092932 PMC3811149

[b27] Park Y. B. . Effects of antirheumatic therapy on serum lipid levels in patients with rheumatoid arthritis: a prospective study. Am J Med. 113, 188–193 (2002). 12208376 10.1016/s0002-9343(02)01186-5

[b28] Kopec G. & Podolec P. Clinical significance of measuring inflammatory markers in patients with pulmonary arterial hypertension. Pol Arch Med Wewn 125, 215–216 (2015). 25827788

[b29] Zabini D. . Comprehensive analysis of inflammatory markers in chronic thromboembolic pulmonary hypertension patients. Eur Respir J. 44, 951–962 (2014). 25034560 10.1183/09031936.00145013

[b30] Quarck R., Nawrot T., Meyns B. & Delcroix M. C-reactive protein: a new predictor of adverse outcome in pulmonary arterial hypertension. J Am Coll Cardiol. 53, 1211–1218 (2009). 19341863 10.1016/j.jacc.2008.12.038

[b31] Cybulska B. . Polish forum for prevention guidelines on dyslipidaemia. Kardiol Pol. 66, 1239–42 (2008). 19105106

[b32] Catapano A. L. . ESC/EAS Guidelines for the Management of Dyslipidaemias: The Task Force for the Management of Dyslipidaemias of the European Society of Cardiology (ESC) and European Atherosclerosis Society (EAS) Developed with the special contribution of the European Assocciation for Cardiovascular Prevention & Rehabilitation (EACPR). Eur Heart J. 37(39), 2999–3058 (2016).27567407

[b33] Miura M. . Effect of Statin Treatment and Low-Density Lipoprotein-Cholesterol on Short-Term Mortality in Acute Myocardial Infarction Patients Undergoing Primary Percutaneous Coronary Intervention - Multicenter Registry From Tokyo CCU Network Database. Circ J. 80, 461–468 (2016). 26658282 10.1253/circj.CJ-15-0889

[b34] Nozue T. Low-Density Lipoprotein Cholesterol Level and Statin Therapy in Patients With Acute Myocardial Infarction (Cholesterol Paradox). Circ J. 80, 323–324 (2016). 26701184 10.1253/circj.CJ-15-1320

[b35] Weverling-Rijnsburger A. W., Jonkers I. J., van Exel E., Gussekloo J. & Westendorp R. G. High-density vs low-density lipoprotein cholesterol as the risk factor for coronary artery disease and stroke in old age. Arch Intern Med. 163, 1549–1554 (2003). 12860577 10.1001/archinte.163.13.1549

[b36] Zeljkovic A. . LDL and HDL subclasses in acute ischemic stroke: prediction of risk and short-term mortality. Atherosclerosis 210, 548–554 (2010). 20022325 10.1016/j.atherosclerosis.2009.11.040

[b37] Rauchhaus M. . The relationship between cholesterol and survival in patients with chronic heart failure. J Am Coll Cardiol 42, 1933–1940 (2003). 14662255 10.1016/j.jacc.2003.07.016

[b38] Anand I. S. . C-reactive protein in heart failure: prognostic value and the effect of valsartan. Circulation. 112, 1428–1434 (2005). 16129801 10.1161/CIRCULATIONAHA.104.508465

[b39] Harris H. W., Grunfeld C., Feingold K. R. & Rapp J. H. Human very low density lipoproteins and chylomicrons can protect against endotoxin-induced death in mice. J Clin Invest. 86, 696–702 (1990). 2394827 10.1172/JCI114765 PMC296783

[b40] Gambardella J. & Santulli G. Integrating diet and inflammation to calculate cardiovascular risk. Atherosclerosis 253, 258–261 (2016). 27594541 10.1016/j.atherosclerosis.2016.08.041 PMC5813683

[b41] Jasiewicz M., Knapp M., Waszkiewicz E., Musiał W. J. & Kamiński K. A. Potential pathogenic role of soluble receptor activator of nuclear factor-ĸB ligand and osteoprotegerin in patients with pulmonary arterial hypertension. Pol Arch Med Wewn 124, 579–586 (2014). 25188298 10.20452/pamw.2491

[b42] Pfister S. L. & Campbell W. B. Reduced pulmonary artery vasoconstriction in methacholine in cholesterol-fed rabbits. Hypertension 27, 804–810 (1996). 8613244 10.1161/01.hyp.27.3.804

[b43] Lourenço A. P. . A Western-type diet attenuates pulmonary hypertension with heart failure and cardiac cachexia in rats. J Nutr. 141, 1954–1960 (2011). 21940516 10.3945/jn.111.145763

[b44] Rysz-Górzynska M. . Efficacy of Statin Therapy in Pulmonary Arterial Hypertension: A Systematic Review and Meta-Analysis. Sci Rep. 22, 30060 (2016). 10.1038/srep30060 PMC4957081 27444125

[b45] Zdrojewski T. . Prevalence and control of cardiovascular risk factors in Poland. Assumptions and objectives of the NATPOL 2011 Survey. Kardiol Pol. 71, 381–392 (2013). 23788344 10.5603/KP.2013.0066

[b46] Zahorska-Markiewicz B. . Polish Forum for Prevention Guidelines on overweight and obesity. Kardiol Pol. 66, 594–6 (2008). 18630394

[b47] Kopeć G. Atherosclerosis progression affects the relationship between endothelial function and aortic stiffness. Atherosclerosis. 204, 250–4 (2009). 18922528 10.1016/j.atherosclerosis.2008.09.003

[b48] American Diabetes Association. 2. Classification and Diagnosis of Diabetes. Diabetes Care. 39, 13–22 (2016). 26696675 10.2337/dc16-S005

